# Study of serum visfatin and blood glucose and lipid metabolism, nafld in simple obese children

**DOI:** 10.1186/1687-9856-2015-S1-P74

**Published:** 2015-04-28

**Authors:** Rui-zhen Li, Hui-ling Lu, Xin-yu Ma, Shi-xiu Kang

**Affiliations:** 1 Children’s Hospital, Wuhan, China; 2 TongJi Hospital Affiliated to TongJi Medical College of Huazhong University of Science and Technology, Wuhan, China

**Keywords:** Visfatin, Obesity, Children, Fatty liver

## Aims

To investigate the relationships between serum visfatin and blood glucose and lipid metabolism, nonalcoholic fatty liver disease (NAFLD) in simple obese children.

## Methods

Eighty-six children (57 boys and 29 girls) including 40 obese children, 22 overweight children and 24 healthy children were recruited with ages ranged 7-15 years.The serum visfatin levels were determined by ELISA.

## Results

(1) As compared with normal and overweight children, serum visfatin levels increased 49.80%( P<0.05) and 35.88% (P<0.05); There were no difference of serum visfatin levels between the healthy and overweight children. (2) The body mass index, TC, TG, LDL-c, FPG, FINS and insulin resistance index of the obese children were higher than the healthy children (p<0.05 or p<0.01); the HDL-c and insulin sensitivity index of the obese children were lower than the healthy group (p<0.01 or p<0.05).The body mass index, the fasting insulin and the insulin resistance index of the obese children were higher than the overweight children (p<0.01).The ALT and AST levels of the obese children were higher than the healthy group (p<0.05).There was significant difference between the obese children and healthy children on the no-alcoholic fatty liver disease (p<0.01) with the prevalence of 23.33% and 5.26%, respectively.(3) Correlation analysis: Body mass index, TG and ALT were positively correlated serum visfatin (r=0.218, p<0.05; r=0.500, p<0.01; r=0.426, p<0.01, respectively).

## Conclusions

The serum visfatin level is correlated with obesity and it is related with the disorder of lipid metabolism and the occurrence of fatty liver in obese children.Serum visfatin levels can be used as a new indicator to evaluate the trend of childhood obesity and valuate the risk of fatty liver, diabetes and cardiovascular of the obese children in the future.

